# More *Cercospora* Species Infect Soybeans across the Americas than Meets the Eye

**DOI:** 10.1371/journal.pone.0133495

**Published:** 2015-08-07

**Authors:** Ana Paula Gomes Soares, Eduardo A. Guillin, Leandro Luiz Borges, Amanda C. T. da Silva, Álvaro M. R. de Almeida, Pablo E. Grijalba, Alexandra M. Gottlieb, Burton H. Bluhm, Luiz Orlando de Oliveira

**Affiliations:** 1 Departamento de Bioquímica e Biologia Molecular, Universidade Federal de Viçosa, Viçosa (MG), Brazil; 2 Instituto de Genética Ewald A. Favret, CNIA, INTA Castelar, Buenos Aires, Argentina; 3 Empresa Brasileira de Pesquisa Agropecuária, Embrapa-Soja, Londrina (PR), Brazil; 4 Facultad de Agronomía, Universidad de Buenos Aires, Buenos Aires, Argentina; 5 Departamento de Ecología, Genética y Evolución, Facultad de Ciencias Exactas y Naturales, Universidad de Buenos Aires, Buenos Aires, Argentina; 6 Consejo Nacional de Investigaciones Científicas y Técnicas (CONICET), Buenos Aires, Argentina; 7 University of Arkansas, Fayetteville, Arkansas, United States of America; University of Innsbruck, AUSTRIA

## Abstract

Diseases of soybean caused by *Cercospora* spp. are endemic throughout the world’s soybean production regions. Species diversity in the genus *Cercospora* has been underestimated due to overdependence on morphological characteristics, symptoms, and host associations. Currently, only two species (*Cercospora kikuchii* and *C*. *sojina*) are recognized to infect soybean; *C*. *kikuchii* causes Cercospora leaf blight (CLB) and purple seed stain (PSS), whereas *C*. *sojina* causes frogeye leaf spot. To assess cryptic speciation among pathogens causing CLB and PSS, phylogenetic and phylogeographic analyses were performed with isolates from the top three soybean producing countries (USA, Brazil, and Argentina; collectively accounting for ~80% of global production). Eight nuclear genes and one mitochondrial gene were partially sequenced and analyzed. Additionally, amino acid substitutions conferring fungicide resistance were surveyed, and the production of cercosporin (a polyketide toxin produced by many *Cercospora* spp.) was assessed. From these analyses, the long-held assumption of *C*. *kikuchii* as the single causal agent of CLB and PSS was rejected experimentally. Four cercosporin-producing lineages were uncovered with origins (about 1 Mya) predicted to predate agriculture. Some of the *Cercospora* spp. newly associated with CLB and PSS appear to represent undescribed species; others were not previously reported to infect soybeans. Lineage 1, which contained the ex-type strain of *C*. *kikuchii*, was monophyletic and occurred in Argentina and Brazil. In contrast, lineages 2 and 3 were polyphyletic and contained wide-host range species complexes. Lineage 4 was monophyletic, thrived in Argentina and the USA, and included the generalist *Cercospora cf*. *flagellaris*. Interlineage recombination was detected, along with a high frequency of mutations linked to fungicide resistance in lineages 2 and 3. These findings point to cryptic *Cercospora* species as underappreciated global considerations for soybean production and phytosanitary vigilance, and urge a reassessment of host-specificity as a diagnostic tool for *Cercospora*.

## Introduction

Due to a variety of environmental and genetic constraints, the ability of a pathogen to cause disease in a host plant is not the rule but an exception. Nevertheless, historical records of major disease outbreaks, such as the potato blight that caused the infamous Irish famine of the mid-1800s, serve as reminders of the relentless power of plant pathogens. One of the key elements for understanding how plant diseases originate, disseminate, and can be managed is the proper identification of their causal agents.

The cosmopolitan genus *Cercospora* Fresen. (Mycosphaerellaceae, Ascomycota) contains some of the most destructive plant pathogens. Within *Cercospora*, more than 3,000 species have been described [1], although 659 species are currently recognized; 281 morphologically indistinguishable species have been lumped and referred to as *C*. *apii s*. *lat*. [2] after the type species [3]. The lack of a consistent array of diagnostic characters makes species identification in *Cercospora* a daunting task. For most *Cercospora* spp., a sexual stage has never been observed; for the few species with a known sexual stage, the teleomorph falls within the genus *Mycosphaerella*, as demonstrated by molecular phylogenetic analyses [4]. Traditionally, anamorphic members of *Cercospora* have been identified to species based on the morphology of conidia and conidiophores, and on the belief that most species of *Cercospora* are strictly host-specific [4–6], even to the extent that new species have been described solely based upon occurrence in a distinct host [6]. With the advent of molecular tools, a new theme has emerged in *Cercospora* taxonomy: an increasing number of unknown, undescribed species in addition to a number of distinct species with shared symptomatology on a given host [6–8].

According to the current knowledge, there are only two species of *Cercospora* that infect soybeans (*Glycine max* [L.] Merr.): *C*. *sojina* Hara and *C*. *kikuchii* (Tak. Matsumoto & Tomoy.) M.W. Gardner; both are believed to be host specific [9]. While *C*. *sojina* is not known to produce the light-activated, red-purple polyketide toxin cercosporin [5], *C*. *kikuchii* is able to produce and use this toxin as a pathogenicity factor during colonization of soybean seeds and leaves [10]. Despite the difference, a previous phylogenetic study placed both *C*. *sojina* and *C*. *kikuchii* within a cercosporin-producing clade of *Cercospora* species; however, the teleomorphs of both species remain unknown [5].

In soybeans, *C*. *kikuchii* causes Cercospora leaf blight (CLB) and purple seed stain (PSS) [11, 12]. Identification of this pathogen relies heavily on the classical symptoms of PSS: the presence of purple-stained spots against the natural color of the soybean seed coat [11]. The purple discoloration is caused by the accumulation of cercosporin, which causes membrane damage and cell death [13], subsequently decreasing grain marketability and seedling vigor [14]. In addition, *C*. *kikuchii* also infects other aerial parts of the plant, such as the hypocotyls, stems, leaves, and petioles; the disease is then called CLB [12]. Cercospora leaf blight causes significant yield loss when blighting leads to defoliation at the time pods are filling [15]. The distribution of *C*. *kikuchii* is worldwide [16], including major soybean producing regions of USA, Brazil, and Argentina–the top three soybean producing countries [17].

Several lines of evidence suggest etiological anomalies in CLB and PSS. In population genetic studies, an array of markers–AFLP, RAPD, and DNA sequences from a single or few loci–suggested remarkable differences in genetic structure among populations, distribution of pathogenicity ‘groups’ or ‘lineages’, and uneven prevalence throughout the Americas [15, 18, 19]; some of those lineages were absent from Japan [20]. Besides, there were conflicting reports on isolate-dependent conditions for sporulation *in vitro* [21], and phenotypic variation among axenic cultures has been observed [18, 22]. A large number of vegetative compatibility groups were uncovered in *C*. *kikuchii* [14]. Considering that this species has arrived relatively recently in the Americas owing to the recent introduction of soybean cultivation, these variations seem unusually large.

From a practical perspective, management of soybean diseases has relied heavily on applications of broad-spectrum chemical fungicides (benzimidazoles and strobilurins, either alone or in combination) (e.g., [23]). Applications of these fungicides may not be intended to control either CLB or PSS directly; nevertheless, the causal agents are often exposed to strong chemical selective pressures, and thus resistance may emerge over time. Some point mutations are known to confer fungicide tolerance. In the nuclear β-tubulin gene, a non-synonymous transversion A→C gives rise to the Glu_198_→Ala_198_ substitution in the tubulin protein, which confers tolerance to benzimidazole in many fungal pathogens [24] including *Cercospora* [25]. In the mitochondrial cytochrome *b* gene, a non-synonymous transversion G→C results in the substitution Ala_143_→Gly_143_; this point mutation confers tolerance to strobilurins [24], and has been documented among *Cercospora* spp. [26]. Hereafter, these substitutions are referred to as ‘mutation E198A’ and ‘mutation A143G’, respectively.

For this study, we assembled several multilocus data sets with DNA sequences obtained from isolates of ‘*C*. *kikuchii*’ obtained from otherwise typically CLB- and PSS-symptomatic plants sampled throughout major soybean growing areas in the Americas–USA, Brazil, and Argentina. Then, we used an array of complementary phylogenetic and phylogeographic tools to address the following four questions: 1) Is *C*. *kikuchii* the single causal agent of CLB and PSS in soybeans or are there additional causal agents? 2) What are the phylogenetic relationships among the causal agents of CLB and PSS, *C*. *sojina*, and other closely-related *Cercospora* species? 3) Have these hidden lineages emerged in response to agriculturally-related events? 4) What can we learn for improving the management of cryptic pathogenic species in a global agriculture system?

## Materials and Methods

### Ethics Statement

This study was carried out with introduced plant pathogens for which no previous approval of the Committee on the Ethics of Animal Experiments of the Federal University of Viçosa was necessary. Field studies did not involve endangered or protected species. No collecting permits were required as these are introduced, alien plant pathogens of cosmopolitan occurrence. When infested soybeans were collected in the field, the owner's permission was obtained.

### Fungal cultures

Fungal isolates used in this study are part of personal culture collections of L. O. Oliveira (Brazil), E. A. Guillin (Argentina), and B. H. Bluhm (USA) and are maintained at their respective institutions. The cultures were obtained from either infected soybean leaves or purple stained seeds; isolation techniques followed protocols that were described elsewhere [6, 18, 22]. The isolate CBS128.27 = CPC 5068 (ex-type of *C*. *kikuchii*) was acquired from CBS-KNAW Fungal Biodiversity Centre (The Netherlands). Overall, isolates associated with CLB and PSS were obtained from all major soybean producing areas in Argentina and Brazil; in the USA, isolates represent the soybean-producing areas in Arkansas.

### DNA extraction, PCR amplification, and sequencing

Fungal mycelia were collected from cultures grown on potato dextrose agar (PDA) [18]. Total genomic DNA was extracted from mycelia using either a method described previously [27] or the Miniprep DNeasy Plant Mini Kit (QIAgen). Polymerase chain reactions (PCR) were performed to amplify seven nuclear gene regions and the mitochondrial (*cyb*) gene region. The seven nuclear gene regions included the five regions previously used to obtain a comprehensive phylogeny of *Cercospora* [6]. The protocols, primers, and PCR conditions to amplify *act*, *cal*, *his*, ITS, and *tef* were essentially those described previously [6]. For the amplification of the *cyb* gene region, we followed the instructions of a previous study [28]. Detailed information on primer design and usage is provided (S1 Table).

Amplification of the *cfp* gene region required the design of new primers, which was accomplished by analyzing the *cfp* gene of *C*. *kikuchii* (GenBank accession number: AF091042) with Primer3 software [29]. The following two primer pairs were designed: F1771 /R2706 and cfp_F1/cfp_R1 (S1 Table). Primer pair F1771/R2706 anneals internally to primer pair cfp_F1/cfp_R1; both primer pairs amplify a DNA fragment that contains the three introns and flanking coding regions of the *cfp* gene. For most of the isolates, efficient amplification was achieved with primer pair F1771/R2706; however, for some isolates, the primer pair cfp_F1/cfp_R1 yielded better results. The amplifications used 150 ng of genomic DNA, 0.4 uM of each primer, 0.2 mM of each dNTP, 1.5 units of Taq DNA polymerase (Phoneutria), 1X buffer IVB (Phoneutria), and 2.0 mM MgCl_2_ in a final volume of 25 μL. The PCR profile when using primer pair F1771/R2706 was as follows: 3 min at 94°C followed by 30 cycles of 30 s at 94°C for template denaturation, 30 s at 56°C for primer annealing, and 1 min at 72°C for primer extension, with a final extension step of 10 min at 72°C. For primer pair cfp_F1/cfp_R1, the PCR profile was 2 min at 94°C followed by 30 cycles of 45 s at 94°C, 45 s at 58°C, and 45 s at 72°C, with a final extension step of 10 min at 72°C.

Amplification of the *tub* gene region targeted two non-overlapping fragments. Hereafter, we will refer to these fragments as tub_1 and tub_2, respectively. To amplify tub_1, we designed the new primer Ck_Betatub-F1 (S1 Table) by analyzing the complete sequence of the *tub* gene from *C*. *beticola* isolate C-3 (GenBank accession number: AY856373) with Primer3. Reactions utilizing Ck_Betatub-F1 with primer Bt516R [25] yielded tub_1, which contained the first four introns of the *tub* gene. An additional pair of primers–Tub-F1/Tub-R1; from [30]–was used to amplify tub_2, which resided completely within exon 5 of the *tub* gene. For the primer pair Ck_Betatub-F1/Bt516R, we use an annealing temperature of 67°C and for the primer pair Tub-F1/Tub-R1, we use 56°C; for the primer pair Tub-F1/Tub-R1 the PCR profile was identical to that used for primer pair F1771/R2706.

Sequencing services were performed by Macrogen Inc., South Korea (www.macrogen.com), and the University of Arkansas DNA Resource Center (Fayetteville, AR). Sequencing reactions were carried out with the same primers as in the PCR amplifications.

### Sequence alignments and analyses of recombinants

All sequences were imported into Sequencher version 4.8 (Gene Codes Corp.) for editing. Complete sequence alignments were performed with the introduction of gaps to compensate for the presence of insertion/deletions (indels). After all of the sequences were aligned, the ends of the alignments were trimmed to eliminate fragments that could not be obtained for all sequences. For the *tub* region, we concatenated tub_1 and tub_2. Sequences obtained for this study were deposited in GenBank (S2 Table).

Preliminary analyses showed that the *cfp* and *tub* sequences each fell within one of four distinct subsets of sequences. Therefore, we prepared a concatenated *cfp*–*tub* alignment and searched for the presence of intergenic recombinants, that is, isolates that could harbor unexpected *cfp*–*tub* gene combinations. We then scanned the datasets of both *cfp* and *tub* independently with the program RDP3 [31] to search for intragenic recombination events using seven different recombination detection methods.

### Assembly of datasets

Multiple datasets were assembled throughout the study to accommodate the particular requirements of each analysis. From GenBank, we obtained a sequence of *tub* (AY856373) from *C*. *beticola* isolate C-3. Sequences for the remaining five nuclear genes (*act*, *cal*, *his*, ITS, and *tef*) came from the isolate CPC 11557, which is the strain type of *C*. *beticola* [6]. The six sequences of *C*. *beticola* were concatenated and included together with our own set of corresponding sequences to form dataset S1 (N = 27; 3615 bp). In addition to *C*. *beticola* (used as outgroup), therefore, dataset S1 contained 25 fungal isolates associated with CLB and PSS, one isolate of *C*. *sojina* and one isolate of *C*. *beticola*. The sequences were obtained from fungal isolates from Argentina (N = 7), Brazil (11), USA (6), and CPC 5068, the ex-type of *C*. *kikuchii*.

This dataset was intended to explore the phylogenetic relationships among species of *Cercospora* that infect soybeans. Dataset S1a (N = 82; 714 bp) was a variant of dataset S1 and contained sequences for tub_2 only. An isolate from Argentina, Viki_2_2, lacked the sequence for tub_2 and therefore was removed from this analysis. In addition to the set of 26 sequences generated during this study, this dataset was supplemented with 56 sequences available from GenBank for tub_2 [20]. The Imazaki’s collection [20] included isolates from Argentina (29), Brazil (4), and Japan (23); most of these isolates were obtained from 2001 to 2004.

Dataset S2 (N = 60; 1566 bp) contained sequences from a much broader sample of congeneric species. Because sequences of *cfp* and *tub* were not available for all species, we restricted this dataset to sequences of the five nuclear genes (act, *cal*, *his*, ITS, and *tef*) [6] together with our own set of corresponding sequences. The sequences belonged to 18 recognized species of *Cercospora*, with *C*. *apii* as outgroup. Dataset S2 aimed to uncover phylogenetic relationships among soybean-infecting *Cercospora* and other congeneric species within a broader phylogeny of the genus that has been recently published [6].

Dataset S3 (N = 13; 3371 bp) was a smaller version of dataset S1 and was assembled based on the information derived from the previous phylogenetic analyses. Dataset S3 contained sequences of fungal isolates associated with CLB and PSS (11 sequences), *C*. *sojina* (1), and *C*. *beticola* as an outgroup. For *tub*, we split the sequence information into exons (TUBexon) and introns (TUBintron) and considered each subset as a separate partition. This dataset was used for time divergence estimates.

We also assembled five additional datasets that were intended for network analyses. Four datasets were created containing the concatenated sequences of the *cfp* and *tub* genes; there was one dataset for each subset harboring the expected *cfp*–*tub* combination: dataset S4 (N = 54; 2043 bp), S5 (N = 12; 2042 bp), S6 (N = 8; 2049 bp), and S7 (N = 23; 2042 bp). Finally, we assembled dataset S8 for the mitochondrial *cyb* gene region (N = 105; 653 bp); this dataset included sequences from isolates harboring NR. In datasets S4 to S8, each indel, regardless of its size, was considered a fifth state and coded as a single mutation.

### Bayesian phylogenies

The phylogenetic relationships among the species of *Cercospora* were inferred by means of Bayesian phylogeny. Subsets of sequences of each of the seven nuclear gene regions were input independently into the software MRMODELTEST v.2.3 [32]. For each gene region, the Akaike Information Criterion [33] indicated the best-fit models among 24 models of molecular evolution as following: GTR+G (*act*), GTR+I (*cal*, *his*, ITS, and *tef*), HKY+G (*cfp*), and GTR+I+G (*tub*). The partitioned analysis was performed in MRBAYES v3.1.2 [34] with dataset S1. The analysis was carried out using two simultaneous runs of five million generations each, with one cold and seven heated chains in each run; the temperature parameter was set to 0.25 (Temp = 0.25) and the branch length prior mean was set to 0.01 (brlenspr = Unconstrained:Exponential(100)). Trees were sampled once every 5,000 generations.

A second phylogenetic analysis investigated the relationships of *Cercospora* and 18 closely-related, congeneric species. The partitioned analysis was performed in MRBAYES v3.1.2 with Dataset S2 and the following models: HKY+G (*act*), GTR+G (*cal*), GTR+I (*his*), GTR+I (ITS), and GTR+I+G (*tef*). This analysis was carried out using two simultaneous runs of 20 million generations each, with one cold and seven heated chains in each run; the temperature parameter was set to 0.25 and the branch length prior mean was set to 0.01. Trees were sampled once every 20,000 generations.

In the preceding analyses, the first 250 trees were discarded as burn-in samples; average standard deviation of split frequencies at the end of each run was below 0.01. The selected settings ensured sufficient sampling of the posterior occurred; in Tracer 1.5 [35], the Effective Sample Size, ESS, values were well above 400 for all statistics. For each analysis, a 50%-majority-rule consensus tree of the two independent runs was obtained with posterior probabilities that were equal to bipartition frequencies

The third phylogenetic analysis compared the information we have gathered in our collection of isolates with the results of a previous investigation on molecular diversity of *C*. *kikuchii* [20]. This analysis was performed in MRBAYES V3.1.2 with Dataset S1a, having GTR+I+G as the model of molecular evolution. The analysis was carried out using two simultaneous runs of one million generations each, with one cold and seven heated chains in each run; the temperature parameter was set to 0.25 (Temp = 0.25). Trees were sampled once every 1,000 generations; the first 250 trees were discarded as burn-in samples; average standard deviation of split frequencies at the end of each run was 0.01 and ESS values were well above 200 for most statistics. A 50%-majority-rule consensus tree of the two independent runs was obtained with posterior probabilities that were equal to bipartition frequencies.

### Divergence dating

The time of divergence among lineages of *Cercospora* associated with CLB and PSS was estimated with BEAST v1.7.5 [35] with sequence information from dataset S3. With the help of BEAUTY v1.7.5 [35], a XML-formatted input file was created for BEAST. In this input file, the substitution models and clock models were unlinked. For each gene partition, we implemented HKY as the model of molecular evolution. As no fossil records exist to calibrate the phylogeny, we relied upon the molecular clock hypothesis and used published mutation rates for the exons and introns of the tubulin gene as previously calculated [36]. We assumed that the mutation rates of the TUBexon and TUBintron partitions followed a normal distribution, with mean = 1.59 (SD = 0.35) substitutions per site per million year (s/s/mi) and 7.46 (SD = 2.0) s/s/mi, respectively. At these rates, introns mutate 4.7 times faster than exons at the *tub* region [36]. The analysis was carried out under the strict molecular clock assumption. A Yule speciation process model was selected for tree prior. The analysis was run for 10 million generations, with samples taken every 10.000 generations. These settings ensured that both model parameters and time estimates were sampled adequately, as the ESS values were above 400 for all statistics in Tracer 1.5. Means and 95% highest posterior density (HPD) intervals were determined in Tracer 1.5. The maximum clade credibility tree with posterior probability of each node was generated with TREEANOTATOR v2.1.2 [35], with a burn-in of 25%.

### Network analyses and neutrality test statistics

Gene genealogies were inferred independently using the median-joining (MJ) network method [37] as implemented in NETWORK 4.6.1.1 (Fluxus Technology Ltd) with default parameters. To infer genetic connections in the network analyses, we used information from the concatenated sequences of the nuclear cfp and tub genes (datasets S4 to S7) and the mitochondrial *cyb* gene (dataset S8). Measures of molecular diversity (number of haplotypes, H; haplotype diversity, H_d_; nucleotide diversity, pi; average number of nucleotide diversity, k; and number of variable sites, S) were estimated in DNAsp v5 [38]. The Tajima’s D [39] test of selective neutrality was carried out in DNAsp v5.

### Amino acid substitutions

Sequence alignments were visually inspected for the occurrence of amino acid substitutions known to confer resistance to fungicides. In the *tub* gene–datasets S4 to S7, we searched for the mutation E198A. We also recorded the occurrences of mutation E198A in the sequences of 56 isolates described previously [20]. In the *cyb* gene–dataset S8, we searched for the mutation A143G. We recorded which isolates harbored any of these two mutations and investigated how these mutations related to other molecular variation in a context of haplotype networks.

### Cercosporin production assay

The concentration of cercosporin was determined in six isolates using the method of [40] following the protocols described in [18].

## Results

### Exploratory analyses: Evidence for recombination

We successfully obtained partial sequences of the β-tubulin 1 (*tub*) and cercosporin facilitator protein (*cfp*) genes from 45 isolates from Argentina, 40 isolates from Brazil, and 22 isolates from the USA, in a total of 107 isolates (Table 1). Analyses revealed a remarkable pattern of genetic variation, with sequences grouping into four subsets and showing a highly congruent distribution for the *cfp* and *tub* genes (S2 Table). Nevertheless, 12 isolates exhibited incongruent *cfp*–*tub* gene combinations. Nine of these (eight from Brazil and one from Argentina) exhibited a pattern that combined a sequence belonging to *cfp* subset 1 with *tub* subset 2. Two additional isolates from Brazil (Ck_A103 and Ck_A114) and one from Argentina (V2_8) displayed a complex recombination pattern. The RDP3 analysis searched for recombination events within each gene region. For the *cfp* gene, there was no evidence for recombination; for the *tub* gene, there was evidence for two independent recombinant events (S2 Table). The 12 recombinant sequences contained eight haplotypes and exhibited high values of nucleotide diversities (Table 2); these sequences were thus excluded from subsequent phylogenetic and phylogeographic analyses.

**Table 1:** Origin of the isolates of *Cercospora* associated with purple seed stain in soybeans. Countries where samples were obtained for DNA sequencing of seven nuclear gene regions (*cfp*, *tub*, *act*, *cal*, *his*, ITS, and *tef*,) and one mitochondrial gene region (*cyb*) are listed, together with the number of isolates successfully sequenced per gene region.

Fungal isolates	Gene region*							
	*cfp*	*tub*	*act*	*cal*	*his*	ITS	*tef*	*cyb*
Full dataset	107	107	23	22	24	24	23	105
Argentina	45	45	7	7	7	7	7	44
Brazil	40	40	11	9	11	11	10	39
USA	22	22	5	6	6	6	6	22

*Gene codes: *cfp*, cercosporin facilitator protein; *tub*, β-tubulin 1; *act*, actin; *cal*, calmodulin; *his*, histone H3; ITS: internal transcribed spacers and intervening 5.8S nrDNA; *tef*, translation elongation factor 1-alpha; *cyb*, cytochrome b.

**Table 2:** Measures of nucleotide diversities and neutrality test statistics for the cercosporin facilitator protein (*cfp*) and β-tubulin 1 (*tub*) gene regions of *Cercospora* associated with purple seed stain in soybeans.

Groups	Sample size (# of haplotypes—h)	Haplotype diversity (H_d_)	Nucleotide diversity (pi)	Average # of nucleotide differences (k)	Number of variable sites (S)	Tajima’s D (*P*-value)
*cpf* ^*a*^ ^,^ ^b^	96 (15)	0.76	0.087	74.0	210	
Lineage 1^b^	54 (2)	0.33	0.0004	0.33	1	0.66 (>0.10)
Lineage 2	12 (4)	0.56	0.0009	0.80	4	-1.38 (>0.10)
Lineage 3	8 (3)	0.46	0.0006	0.50	2	-1.31 (>0.10)
Lineage 4	22 (6)	0.54	0.0007	0.62	5	-1.63 (>0.10)
*tub* ^*a*^ ^,^ ^b^	96 (28)	0.75	0.012	15.3	100	
Lineage 1^a^	54 (6)	0.24	0.0008	1.03	26	-2.66 (<0.001)
Lineage 2	12 (6)	0.88	0.0067	8.04	19	1.21 (>0.10)
Lineage 3	8 (8)	1.00	0.0074	8.82	24	-0.45 (>0.10)
Lineage 4^b^	22 (8)	0.79	0.0034	4.03	25	-1.56 (>0.10)
Concatenated set^a^ ^,^ ^c^	108 (44)	0.86	0.045	91.9	316	
Lineage 1^a^	54 (8)	0.46	0.0007	1.36	27	
Lineage 2	12 (8)	0.92	0.0043	8.85	23	
Lineage 3	8 (8)	1.00	0.0046	9.32	26	
Lineage 4^b^	22 (12)	0.90	0.0023	4.65	30	
Recombinants	12 (8)	0.85	0.0124	25.39	146	

^a^ with the addition of sequence data from CPC 5068, the type strain of *C*. *kikuchii*.

^b^ excluding 12 sequences from recombinant isolates (harboring *cfp* from Lineage 1 and *tub* from Lineage 2).

^c^ including 12 sequences from recombinant isolates (harboring *cfp* from Lineage 1 and *tub* from Lineage 2).

### Molecular variation in *cfp* and *tub* genes

Among the 96 isolates that showed no evidence for recombination, there were 15 haplotypes of the *cfp* gene and 28 for the *tub* gene (Table 2). Four sequence datasets were built and analyzed; nucleotide diversity indices agreed in confirming that levels of genetic variation were concentrated among rather than within subsets. Hereafter, a subset of sequences is referred to as a genealogical lineage (see Bayesian Phylogeny section below). Most of the polymorphisms among the four lineages were concentrated within introns, which included indels, making sequence alignments difficult to achieve (data not shown). Tests of selective neutrality (Table 2) recovered non-significant values for Tajima’s D (P > 0.10) for most lineages and supported the null hypothesis of a neutrally evolving population. Lineage 1 of the *tub* gene was the only exception; the significant negative value for Tajima’s D (-2.66; P < 0.001) indicated an excess of low frequency polymorphism and is an indicative of either population expansion or purifying selection. Therefore, the patterns of distribution of nucleotide variation in both *cfp* and *tub* genes suggested these fungal isolates could be grouped into four genealogical lineages.

### Four lineages associated with CLB and PSS

To question whether the four genealogical lineages would hold when considering a broader sample of the nuclear genome, we analyzed sequence information from five additional nuclear genes–actin (*act*), calmodulin (*cal*), histone H3 (*his*), internal transcribed spacers and intervening 5.8S nrDNA (ITS), and the translation elongation factor 1-alpha (*tef*) gene regions (Table 1).

**Fig 1:**
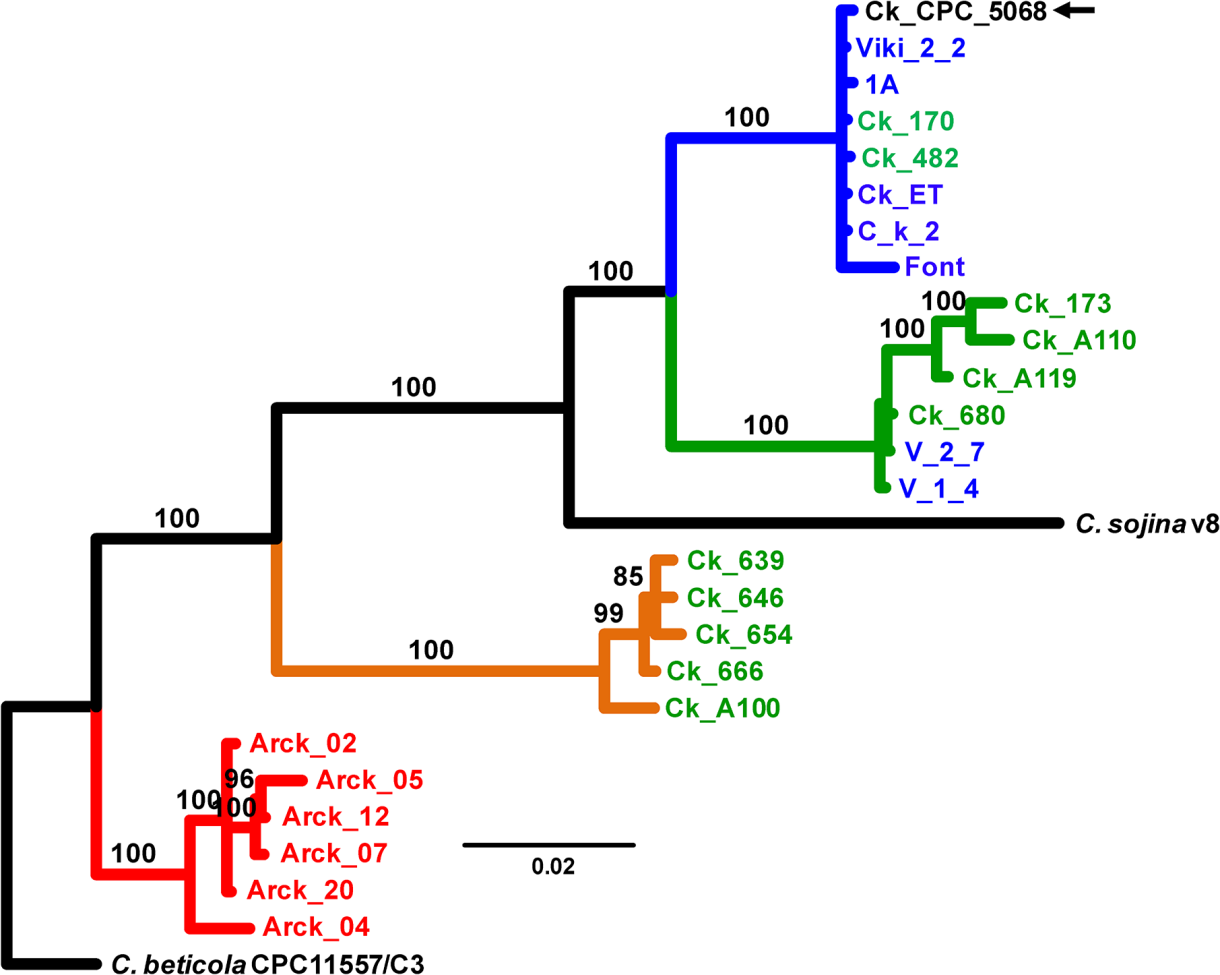
Bayesian phylogeny (consensus tree) showing the relationships among causal agents of Cercospora leaf blight and purple seed stain of soybean. The dataset was 3615 bases long and resulted from the concatenation of seven nuclear genes (*act*, *cal*, *his*, ITS, *tef*, *cfp*, and *tub*). *Cercospora sojina* is shown for reference purpose; the tree was rooted in *C*. *beticola*. Branch lengths are drawn to scale; nodal support values are given as posterior probabilities (%) above the branches (when ≥85%). Scale bar corresponds to the expected number of substitutions per site. Color in terminals according to the origin of the isolates: blue, Argentina; green, Brazil; red, United States. Color in branches according to lineage: blue, lineage 1; green, lineage 2; orange, lineage 3; and red, lineage 4. Black arrow indicates the ex-type strain of *C*. *kikuchii* (CPC_5068). Gene codes: *act*, actin; *cal*, calmodulin; *his*, histone H3; ITS: internal transcribed spacers and intervening 5.8S nrDNA; *tef*, translation elongation factor 1-alpha; *cfp*, cercosporin facilitator protein; *tub*, β-tubulin 1.

A Bayesian phylogeny considering all seven nuclear loci inferred the phylogenetic relationships among isolates associated with CLB and PSS, including *C*. *sojina*, utilizing *C*. *beticola* as an outgroup (Fig 1). The Bayesian tree displayed a well-supported topology, with 100% support from posterior probabilities (PP) for all five major clades. The first clade contained the ex-type of *C*. *kikuchii* together with isolates from Argentina (5) and Brazil (2); this clade corresponded to lineage 1. Sister to the latter, there was a clade comprising six isolates (Argentina, 2; Brazil, 4) that belonged to lineage 2. Surprisingly, these clades had *C*. *sojina* as a sister group. A third clade contained exclusively Brazilian isolates that correspond to lineage 3. The fourth major clade comprised six isolates from USA (all belonging to lineage 4), and exhibited a sister relationship to the remaining clades.

The concentration of cercosporin was determined with a spectrophotometric assay (expressed in nmol/mL) in two isolates of lineage 1 (Ck_170, 23.4; Ck_482, 1.62), one isolate of lineage 2 (Ck_A119, 0.98), two isolates of lineage 3 (Ck_654, 1.39; Ck_666, 0.54), and one recombinant (isolate Ck_178, 1.76). Isolates of lineage 4 also produced cercosporin (B. H. Bluhm, unpublished data). These results revealed that all four lineages are among the cercosporin-producing *Cercospora*.

### Phylogenetic placement of the four lineages

**Fig 2:**
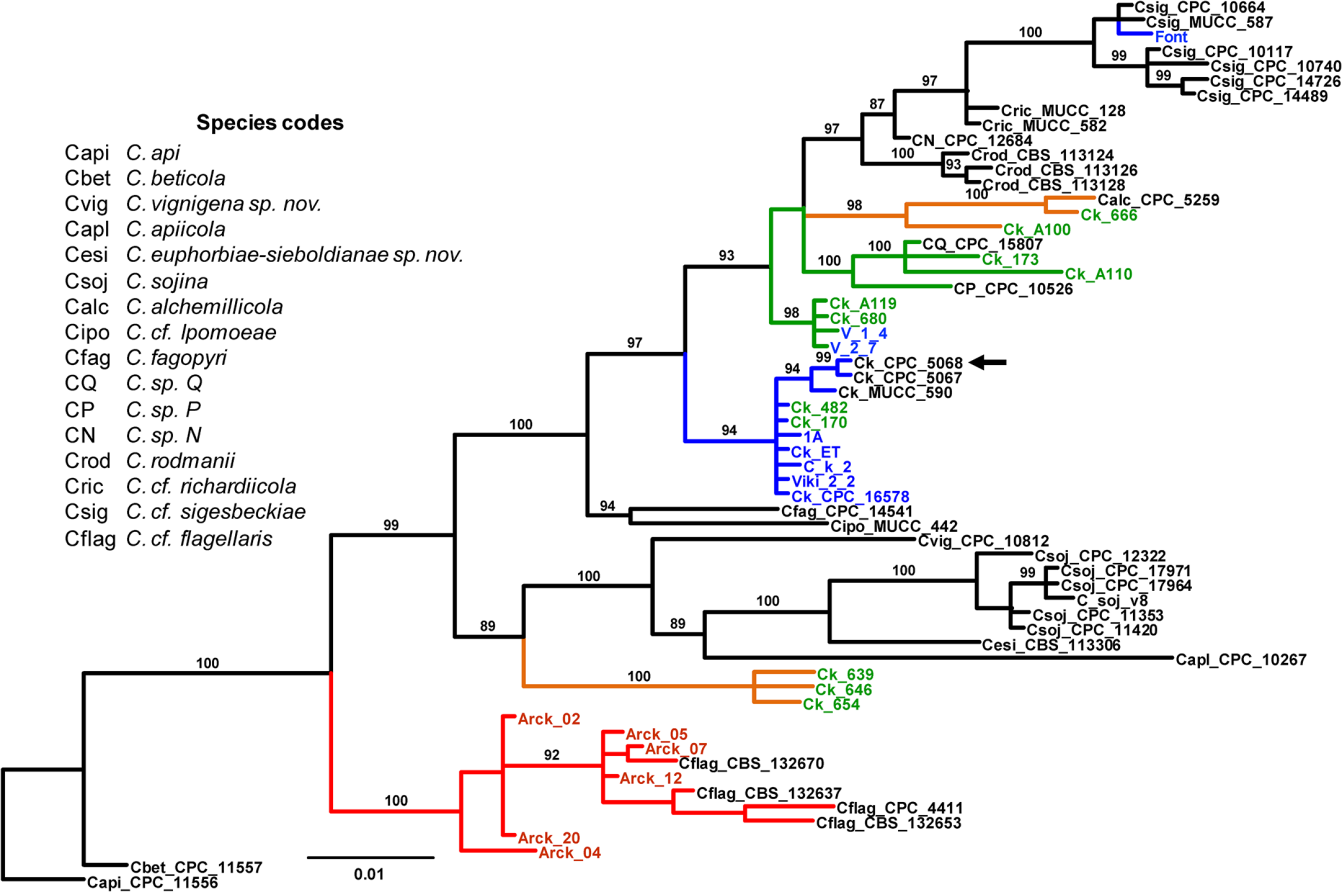
Bayesian phylogeny (consensus tree) showing the relationships among causal agents of Cercospora leaf blight and purple seed stain of soybean and other closely related species. Fungal isolates were obtained from either purple-stained soybean seeds or infected leaves. The dataset was1566 bases long and resulted from the concatenation of five nuclear genes (act, cal, his, ITS, and tef). Branch lengths are drawn to scale; nodal support values are given as posterior probabilities (%) above the branches (when > 85%). Scale bar corresponds to the expected number of substitutions per site. Color in terminals according to the origin of the isolates: blue, Argentina; green, Brazil; red, United States; and black, data from 6]. Color in branches according to lineage: blue, lineage 1; green, lineage 2; orange, lineage 3; and red, lineage 4. Black arrow indicates the ex-type strain of *C*. *kikuchii* (CPC_5068). Gene codes: *act*, actin; *cal*, calmodulin; *his*, histone H3; ITS, internal transcribed spacers and intervening 5.8S nrDNA; *tef*, translation elongation factor 1-alpha.

To assess phylogenetic relationships among the four lineages, we carried out an additional Bayesian phylogeny to place the lineages within a broader phylogenetic context [6], including 18 other species of *Cercospora* (Fig 2). The resulting tree revealed that the four lineages were spread across the phylogeny. Most isolates of lineage 1 formed a monophyletic clade (PP = 94%); the only exception was the Argentinean isolate Font, which nested within a clade of *C*. *cf*. *sigesbechiae* isolates, a species showing a sister relationship with *C*. *kikuchii* [6]. Isolates of lineage 2 did not form a single clade; instead they clustered with two undescribed species and formed sub-clades (PP ≥ 98%). While two members of lineage 2 from Brazil (Ck_173 and Ck_A110) grouped together with *Cercospora sp*. P and *Cercospora sp*. Q, the remaining four members (Ck_A119 and Ck_680, from Brazil; V_1_4 and V_2_7 from Argentina) formed a sister clade. Similarly, isolates of lineage 3 were split into two clades (PP ≥ 98%). Together with *C*. *alchemillicola*, isolates Ck_666 and Ck_A100 formed a clade; the remaining three isolates (Ck_639, Ck_646, and Ck_654) formed a distant clade, sister to the clade that harbors *C*. *sojina*, *C*. *vignigena*, *C*. *euphorbiae-sieboldianae*, and *C*. *apiicola*. All six isolates of *C*. *sojina* formed a monophyletic clade (PP = 100%). Isolates of lineage 4 grouped together with isolates of *C*. *cf*. *flagellaris* in a sister clade (PP = 100%).

### Estimated date of divergence

**Fig 3:**
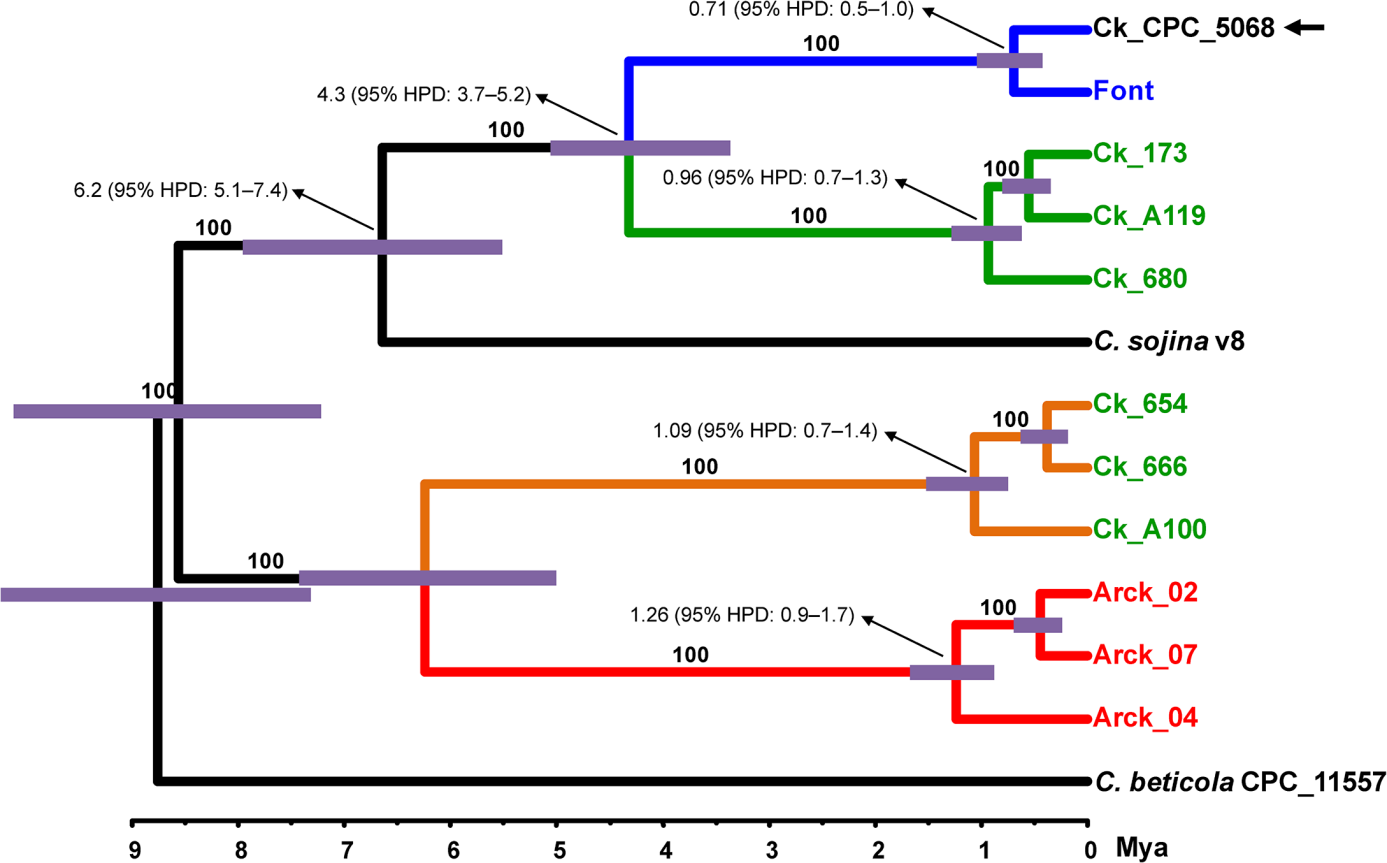
Maximum clade credibility tree for seven nuclear genes of the causal agents of Cercospora leaf blight and purple seed stain of soybean. Scale of the x axis is ages in millions of years (Mya). *Cercospora sojina* is shown for reference purpose; the tree was rooted in *C*. *beticola*. Color in terminals according to the origin of the isolates: blue, Argentina; green, Brazil; and red, United States. Color in branches according to lineage: blue, lineage 1; green, lineage 2; orange, lineage 3; and red, lineage 4. Black arrow indicates the ex-type strain of *C*. *kikuchii* (CPC_5068). Nodal support values are given as posterior probabilities (%) above the branches. Blue bars at nodes are the 95% highest probability density (HPD) intervals for the age of that node.

The BEAST analysis estimated the time of divergence among the four lineages of *Cercospora* and yielded a totally supported maximum clade credibility tree (Fig 3). The results suggested the following placements for the divergences: (a) each of the four lineages started to diverge about 0.5–1.3 Mya; (b) the divergence between the two pairs of sister lineages (1–2, and 3–4) took place much earlier, about 4.3 and 6.2 Mya, respectively; and (c) the ancestor of *C*. *sojina* split from the common ancestor of lineages 1 and 2 about 6.5 Mya.

We assumed the mutation rates for the exonic and intronic regions of the *tub* gene to be 1.59 (SD = 0.35) and 7.46 (SD = 2.0) s/s/mi, respectively. Therefore, it was possible to estimate the mutation rates (given in s/s/mi) in the remaining six nuclear genes: *act* (mean = 5.47, 95% HPD = 3.9–7.4); *cal* (mean = 5.42, 95% HPD = 3.8–6.9); *his* (mean = 2.0, 95% HPD = 1.3–2.7); ITS (mean = 0.5, 95% HPD = 0.2–0.8); *tef* (mean = 2.98, 95% HPD = 2.1–4.0); and *cfp* (mean = 9.90, 95% HPD = 8.1–11.9). The results clearly showed that mutation rates were highly variable among gene regions. The fastest evolving gene (*cfp*) mutated about 20 times faster than the slowest evolving gene (ITS).

### Genealogical networks based on concatenated *cfp*–*tub* genes

**Fig 4:**
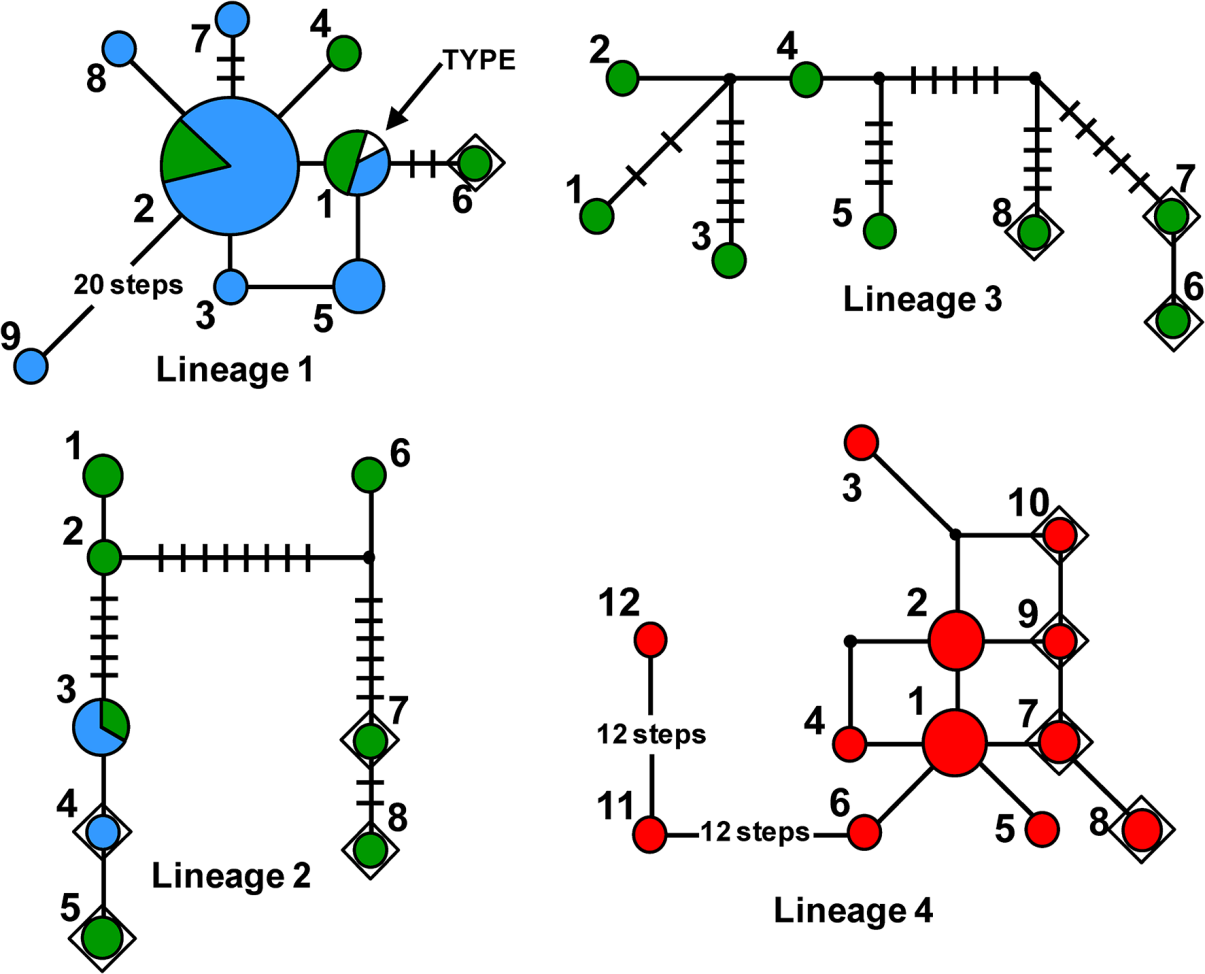
Median-joining networks. Haplotype relationships are depicted within each of the four genealogical lineages of the causal agents of Cercospora leaf blight and purple seed stain of soybean. Networks were based on a dataset containing two nuclear gene regions concatenated: the cercosporin facilitator protein (*cfp*) and the β-tubulin 1 (*tub*) genes. In each network, a circle represents a given haplotype (coded with numbers); size is proportional to the relative frequencies. Numbers of mutational steps are indicated with bars when there is more than one (unless indicated otherwise). Color codes according to the origin of the sequences: blue, Argentina; green, Brazil; and red, United States. Diamonds indicate haplotypes harboring the mutation E198A, which is known to confer resistance to benzimidazole fungicides. In Lineage 1, an arrow denotes the location of the haplotype of CPC 5068, the ex-type strain of *C*. *kikuchii*.

Assessments of the genealogical relationships among haplotypes of the concatenated *cfp*–*tub* gene regions were carried out for each of the four lineages independently (Fig 4), based on the observation that these two gene regions exhibited much higher rates of evolution than the other five nuclear genes. Therefore, we anticipated these networks could reveal important details about the evolutionary history of each of the four lineages. The networks were also anticipated to provide insight about the geographic distribution of the molecular variation in each lineage, including the mutation E198A that is known to confer resistance to benzimidazole fungicides in *Cercospora*.

Lineage 1 exhibited a star-shaped network, which was organized around the most frequent haplotype (haplotype 2), with several low frequency haplotypes occupying tip positions in the network. A single mutation step connected most of the adjacent haplotypes; thus, the arrangement of haplotypes in the network corroborated the lack of resolution within lineage 1 (Figs 1 and 2) and the lower estimates of nucleotide diversities relative to the other three lineages (Table 2). The only exception was the connection between haplotype 2 and haplotype 9 (isolate A2, from Argentina), which took 20 mutation steps. Overall, the topology suggests a rapidly expanding population and is congruent with a significant negative value for Tajima’s D, which suggested population expansion or purifying selection (Table 2). There were only nine haplotypes among 54 sequences; the ex-type of *C*. *kikuchii*, harbored haplotype 1. Only a single isolate–CK_482, from Brazil–displayed the mutation E198A. Lineage 1 was of widespread occurrence both in Argentina and Brazil. Lineages 2 and 3 displayed networks of similar topologies: most of the haplotypes were singletons, that is, the haplotype occurred only once in the dataset; the majority of the connections between two adjacent haplotypes involved a large number of mutational steps, in agreement with the high nucleotide diversity estimates (Table 2); and high frequency of haplotypes harboring the mutation E198A (50% in lineage 2; 37.5% in lineage 3). Even though these lineages contained few members, they were haplotype-rich– 12 sequences gave rise to eight haplotypes in lineage 2, present in both Argentina and Brazil; eight sequences and eight haplotypes are present in Brazilian lineage 3. The network of lineage 4 resembles that of lineage 1. However, the mutation E198A occurred in a much higher frequency in lineage 4; among a total of 22 isolates, six isolates (four haplotypes) bear the mutation. In addition, there were two highly divergent members (haplotypes 11 and 12, containing Arck 05 and Arck 04, respectively). The geographic occurrence of lineage 4 was restricted to USA.

### Genealogical network based on the *cyb* gene

**Fig 5:**
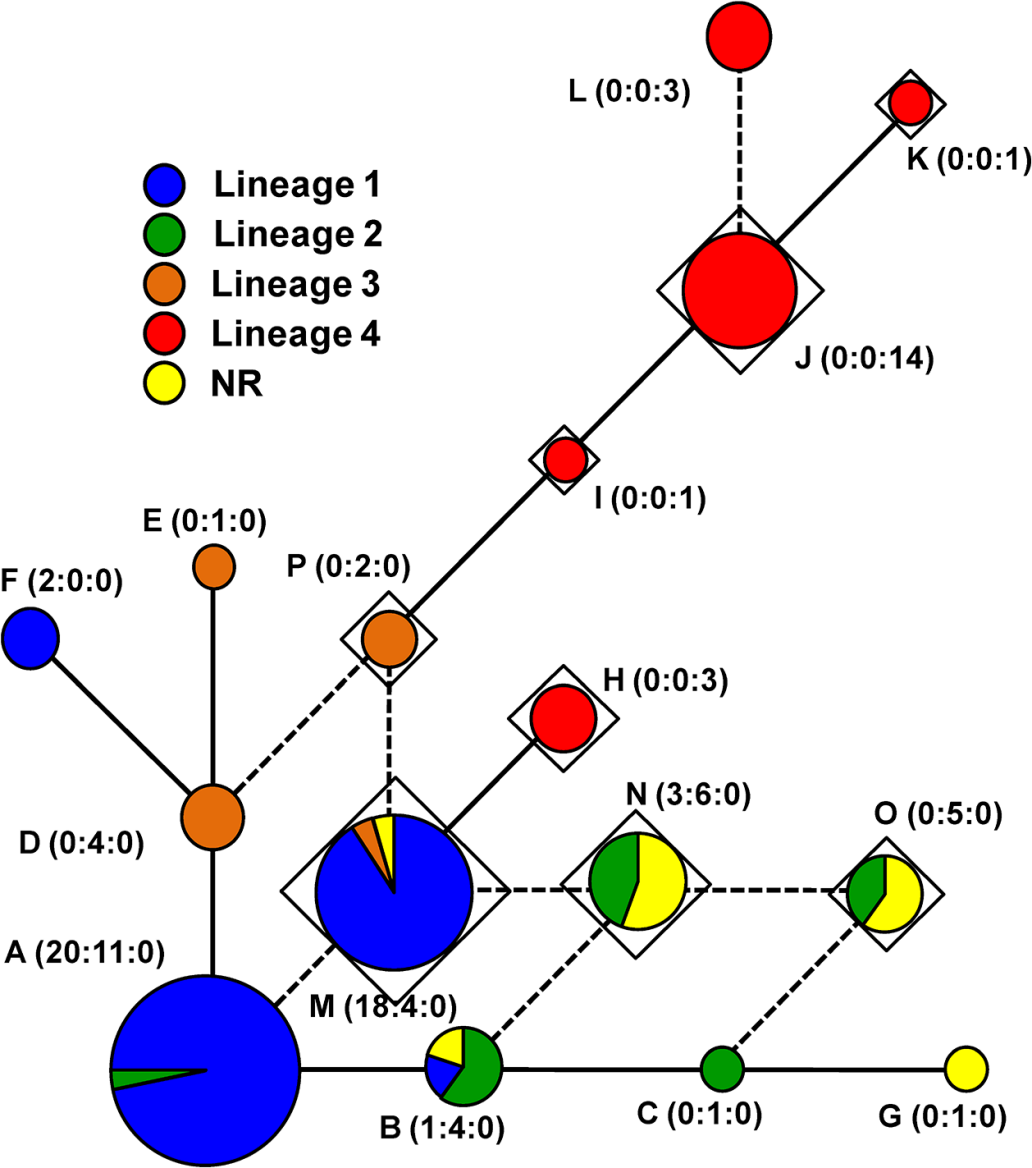
Median-joining network depincting genealogical relationships among mtDNA haplotypes of *Cercospora* that is associated with purple seed stain in soybeans. The network was based on a dataset (653 bases long) obtained from the mitochondrial cytochrome b (*cyb*) gene. Each circle represents a given haplotype (coded with letters); a line linking two haplotypes indicates a single mutation step. Color slices represent the frequencies of that haplotype in each of the four lineages (see Fig 3). NR, isolates harboring unexpected *cfp*–*tub* gene combinations in the nuclear genome. Numbers in parenthesis refer to the number of occurrences of that haplotype in Argentina, Brazil, and USA, respectively. Diamonds indicate haplotypes harboring the mutation A143G, which is known to confer resistance to Qoi inhibitor fungicides. Dashed line indicates network homoplasy owing to multiple, independent origins of mutation A143G.

The genealogical relationships among haplotypes of the mitochondrial cytochrome *b* gene region (*cyb*) were carried out using 105 isolates (Argentina, 44; Brazil, 39; and USA, 22). Therefore, this analysis included mitochondrial sequences from the four lineages previously defined, in addition to sequences of the 12 NR isolates. The 105 sequences were grouped into 16 haplotypes that were not uniformly distributed among the four nuclear lineages (Fig 5).

There was a tendency for mitochondrial haplotypes to group according to their nuclear lineage. To a certain extent, haplotype sharing took place between lineages 1 and 2 (haplotypes A and B) and lineages 1 and 3 (haplotype M). There was no haplotype sharing between lineage 4, geographically restricted to Arkansas–USA, and the lineages that occurred in Argentina and Brazil. Haplotype sharing was most noticeable among isolates of lineage 2 and NR isolates (haplotypes B, N, and O). This sharing of haplotypes suggests that 11 out of 12 NR isolates had inherited the mitochondrial genome from a parental donor that belonged to lineage 2, while receiving part of the nuclear genome from a parental donor of lineage 1.

### The four lineages in Japan

The availability of additional sequences from Imazaki’s collection, comprising tub_2 from Argentina (29), Brazil (4), and Japan (23) [20] allowed us to further explore the geographic distribution of the four lineages. Our main question was whether any of the four lineages occurred in Japan, a source of soybean germplasm for western countries and known for cultivating soybeans for millennia. The resulting tree contained key clades showing high support (PP > 98) (S1 Fig). The apparent difference in the branching pattern with topology in Fig 1 is most likely due to the low number of variable characters in the tub_2 dataset. Nevertheless, the results added relevant information about the geographic distribution of the four lineages that was hidden from our previous analyses. Sequences of the 56 isolates from Imazaki’s collection [20] were spread all throughout the topology, with members in all four lineages. There was clear evidence that all four lineages were present in Japan, with high frequency of lineages 2 and 4. Additionally, this data set indicated that lineage 4 also occurred in Argentina, and that lineage 3 is shared by Argentina and Brazil. A single Japanese isolate (CkJC203) belonged to lineage 1 –the lineage holding the ex-type of *C*. *kikuchii;* additionally, this isolate is unique in displaying the mutation E198A (frequency = 0.017).

## Discussion

The nuclear genes *tub* and *cfp* turned out to be an excellent choice as sources of polymorphic markers to investigate the phylogenetic and phylogeographic relationships among the causal agents of CLB and PSS. Both gene regions contained introns and flanking regions of exons, thus allowing signals of different evolutionary histories, both at the intra- and inter-specific levels, to be retained. Both *cfp* and *tub* harbored a large set of fixed nucleotide positions that easily discriminated our collection of isolates into four genealogical lineages. At the same time, a considerable level of polymorphism within lineages allowed phylogeographic inferences to be made. Therefore, we strongly suggest that future phylogenetic and phylogeographic studies across *Cercospora* incorporate *tub* and *cfp* regions to the set of five nuclear genes currently in use [6].

### Multiple agents cause CLB and PSS

Herein, we provide experimental evidence in support of multiple causal agents of CLB and PSS, contrarily to the current understanding. Phylogenetic analyses show that the multiple *Cercospora* spp. associated with CLB and PSS clustered into four independent lineages, with *C*. *kikuchii* nested within lineage 1. It is very possible that some members of these four lineages represent new species, yet to be described. The estimated dates of divergence among the four lineages (about 1 Mya) suggest that lineage differentiation did not arise due to agriculturally-related events. Most likely, the lineages dispersed from their native Asian territories to the Americas only very recently, owing to human-mediated transport of asymptomatic plant materials. It is somewhat surprising that the configuration of this pathosystem has remained in disguise without being recognized previously. Certainly, one of the most probable reasons that precluded earlier detection was the accepted premise that only two species of *Cercospora* could infect soybeans, namely *C*. *kikuchii* and *C*. *sojina*. The concept that most *Cercospora* spp. are restricted to a narrowly limited host range, even a single host species, was advanced by C. Chupp, an early authority on *Cercospora* [4], and held for decades before the advent of tools for molecular analyses. As a consequence, *C*. *kikuchii* was taken as the single species associated with the easily distinguishable, typical symptoms of PSS or CLB. More recently, studies have split the genetic pool of ‘*C*. *kikuchii*’ into distinct groups [18, 20, 22], but the fingerprinting tools utilized lacked phylogenetic support to allow the recognition of multi-species arrangements within independent genealogical lineages. Additionally, it is likely that previously observed physiological differences among fungal isolates were attributed to individual variability rather than the possible presence of cryptic species. For example, visual assessments of mycelial growth indicated a certain level of morphological variability among members of the four lineages; however, differences observed were not as pronounced as inter-specific differences observed such as between *C*. *sojina* and *C*. *beticola*. In the light of our results, PSS and CLB symptoms on soybeans are complex, homoplasic characters and, as such they can no longer be taken as unambiguous indicators for the presence of *C*. *kikuchii*.

### Monophyly of lineages 1 and 4

Both phylogenetic and network analyses showed compelling evidence to support the monophyly of lineages 1 and 4. Lineage 1 was the most frequent in our collection (57.4%), and contains the type of *C*. *kikuchii*, the species thought to be the single causal agent of CLB and PSS. Further research is needed to rule out the soybean host specificity of lineage 1. Our results confirm the occurrence of *C*. *kikuchii* in Argentina and Brazil; nevertheless, its presence in USA remains uncertain. Analyses of a larger sampling will be necessary to establish whether *C*. *kikuchii* is among the causal agents of CLB and PSS in that country. As to lineage 4, it contains 23 isolates from our collection together with *Cercospora cf*. *flagellaris*. This latter species has been isolated from hosts spread over phylogenetically-unrelated plant families: Amaranthaceae, Asteraceae, Rutaceae, and Salicaceae, to cite a few [6]. This lineage was the second most frequent in our collection (24.5%); its occurrence in Argentina was inferred through the addition of the Imazaki´s collection [20]. The prevalence of this linage in Argentina deserves further scrutiny. In USA, lineage 4 may predominate, as all isolates have that origin. However, as these isolates were collected in a single state (Arkansas), additional sampling effort is needed to survey the other soybean producing states. Our findings urge taxonomic investigations to uncover the true identity of these isolates and to delimit what the species boundaries are, in both Argentina and USA. The apparent absence of lineage 4 from tropical regions of soybean cultivation in Brazil may indicate that this lineage has an ecological preference for colder regions. However, the majority of USA soybean production is concentrated in the Midwest, where the climate is generally cooler than in Arkansas. Thus, the apparent predominance of lineage 4 in Arkansas may be linked to other factors.

### Polyphyly of lineages 2 and 3

Lineage 2 and 3 appear to represent species complexes, with members infecting a wide range of host species, including soybeans. The presence of large gaps showing discontinuity in networks, such as those we uncovered for lineage 2 and 3 (Fig 4), is consistent with long-term barriers to gene flow or limited gene flow among populations [41]. In each lineage, such a scenario is likely if their members evolved on hosts that occupied distinct ecological or geographic ranges for an extended period and later were able to infect soybeans as modern agricultural practices introduced the exotic, susceptible host to their territories. Additional taxonomic and phylogenetic studies are necessary to confirm the extent to which speciation events have occurred and to define species boundaries within lineage 2 and 3. Consistent with the predicted existence of cryptic species of *Cercospora* causing CLB and PSS, the data obtained in this study indicate that lineages 2 and 3 are polyphyletic. Moreover, two Brazilian isolates clustered together with two undescribed species–namely, *Cercospora* sp. Q, and *Cercospora* sp. P–as sister taxa; the host ranges of these two species are wide and encompass a diverse array of phylogenetically-unrelated plant families, including Fabaceae, Euphorbiaceae, Dioscoreaceae, Rutaceae, and Rubiaceae, among others [6]. Brazilian isolates from lineage 3 formed a clade with C. *alchemillicola*, a species reported to infect the ornamental *Alchemilla mollis* (Rosaceae); affine fungal species were also detected in Onagraceae [6]. Notwithstanding, lineages 2 and 3 display each a cluster with isolates obtained from soybeans exclusively.

### Genetic admixture among lineages

The occurrence of genetic admixture was not anticipated. Our results, however, provide evidence suggesting that gene flow occurred among lineages to a certain extent. The large number of nucleotide differences detected among sequences of both *cfp* and *tub* genes indicate that each of these genes underwent distinct evolutionary histories within lineages, and therefore accumulated distinct sets of polymorphisms (mostly within introns, as it would be expected). Our results also supported the occurrence of nuclear genomic recombination, thus providing support for gene flow. Moreover, those recombinants harbor the mitochondrial *cyb* gene from lineage 2, which suggests that the unidirectional inheritance of the mtDNA favored lineage 2 as the donor. We also detected a recombination event between lineage 1 and 3 (isolate A2, from Argentina); isolate A2 can be seen as the divergent haplotype 9 (Fig 4). Likely, recombination events may also explain the origin of the divergent haplotypes 11 and 12 of lineage 4. In this case, the recombination event seems to have taken place between lineage 4 and a parental lineage yet to be known. The mechanism that could allow for admixture among lineages remains elusive, as most species of *Cercospora* have no known sexual stage [5]. However, analyses of mating-type distribution and haplotyping based on microsatellites revealed evidence for cryptic sexual recombination in several species of *Cercospora*, including the soybean pathogen *C*. *sojina* [42, 43], and thus it is plausible that members of one or more of the lineages described in this study are also capable of sexual reproduction. Alternatively, parasexual recombination may account for the observed genetic admixture; up to six vegetative compatibility groups have been reported to exist in ‘*Cercospora kikuchii*’ [14]. The lineages we uncovered and described in this study were defined based on molecular variation of two gene regions only (*cfp* and *tub*). The use of genome-wide marker systems–such as [43]–could reveal the extent to which genetic admixtures took place among lineages, with crucial implications for disease management, plant breeding technologies and fungal taxonomy.

### Implications for disease management

The finding that multiple agents cause CLB and PSS may indicate a need for changes in the way the diseases are managed. In light of the present study, exclusion and eradication tactics need to consider the fact that many species of *Cercospora* that cause CLB and PSS in soybeans could potentially infect a plethora of other hosts. Screening of soybean germplasm for resistance to CLB and PSS has been carried out intensively (e.g, [44, 45]). In USA, few sources of resistance were identified using field trials, including soybean genotypes PI 80837 [46] and cultivar SJ2 [47]. Most likely, this resistance was achieved against lineage 4, the most frequent lineage in USA, and should be tested in Argentina and Brazil against lineage 1 before widespread deployment in plant breeding strategies. The high frequencies of mutations associated with benzimidazole (mutation E198A) and strobilurin fungicides (mutation A143G) in isolates of lineages 2 and 3 are puzzling. They suggest that these fungicides exerted high levels of selective pressure against the pathogen populations, resulting in selection of the resistant individuals and quick buildup of resistant populations in the sites sampled. In contrast, lineage 1 responded differently to the selective pressures, and fungicide resistance is maintained at low frequency. In a context involving cryptic species, such as the one we just described, disease diagnostics based on symptoms alone may trigger species misidentification, which in turn may diminish the importance of phytosanitary measures or delay the elaboration of effective management plans.

The mutation A143G is associated with resistance to strobirulin QoI inhibitor fungicides and should confer an important adaptive advantage in a modern agricultural setting where fungicide application is an essential part of disease management. Multiple, independent origins for the A143G mutation in the mitochondrial genome of CLB- and PSS-associated *Cercospora* and subsequent selection for resistance to fungicide likely occurred in the soybean fields where isolates were collected. This mechanism would account for the origin of the homoplasies in the network (Fig 5; depicted as dashed lines). For example, when the mutation A143G was excluded from network analyses, haplotypes M, N, O, P, and L collapsed into haplotypes A, B, C, D, and J. Haplotypes with the mutation A143G had widespread distribution over the *cyb* network and were found across Argentina, Brazil and USA (Fig 5). Most noticeable was the high frequency of haplotypes of lineage 4 (from USA) and haplotypes of lineage 1 (from Argentina and Brazil) harboring the A143G mutation.

### Cryptic pathogenic species in a global agriculture system

Cryptic species may be frequent in plant pathosystems [48]. There is a recent, growing awareness that cryptic species are an underestimated risk and that recognizing and elucidating cryptic species complex is crucial for food security and phytosanitary vigilance in a globalized agriculture system [6, 7, 49–52]. The study we present herein for a relatively ‘well characterized’ plant disease illustrated how complex a pathosystem’s identity became as data availability on molecular diversity grew in density and geographic scale. The pattern of multi-species arrangements associated with homoplasic symptoms is unlikely to be unique to CLB and PSS or to plant diseases only; therefore, as new models–including animal diseases–are probed within phylogenetic and phylogeographic frameworks, it will become more evident the extent to which this pattern repeats itself.

## Supporting Information

S1 Fig Bayesian phylogeny (consensus tree) based on tub_2 sequences. The dataset was 714 bases. *Cercospora sojina* is shown for reference purpose; the tree was rooted in *C*. *beticola*. Branch lengths are drawn to scale; nodal support values are given as posterior probabilities (%) above the branches (when ≥85%). Scale bar corresponds to the expected number of substitutions per site. Color in ingroup terminals according to the origin of the isolates: blue, Argentina; green, Brazil; red, United States; black, Japan. Color in ingroup branches according to lineage: blue, lineage 1; green, lineage 2; orange, lineage 3; and red, lineage 4. Black arrow indicates the ex-type strain of *C*. *kikuchii* (CPC_5068).
(TIFF)

S1 Table Detailed information on PCR primers used in this study. (PDF)

S2 Table Detailed information on isolates used in this study. (XLS)
