# Supplementary figures and images for: More Cercospora Species Infect Soybeans across the Americas than Meets the Eye
Source: PLoS One. 2015 Aug 7;10(8):e0133495. doi: 10.1371/journal.pone.0133495 (PMC4529236; doi:10.1371/journal.pone.0133495)

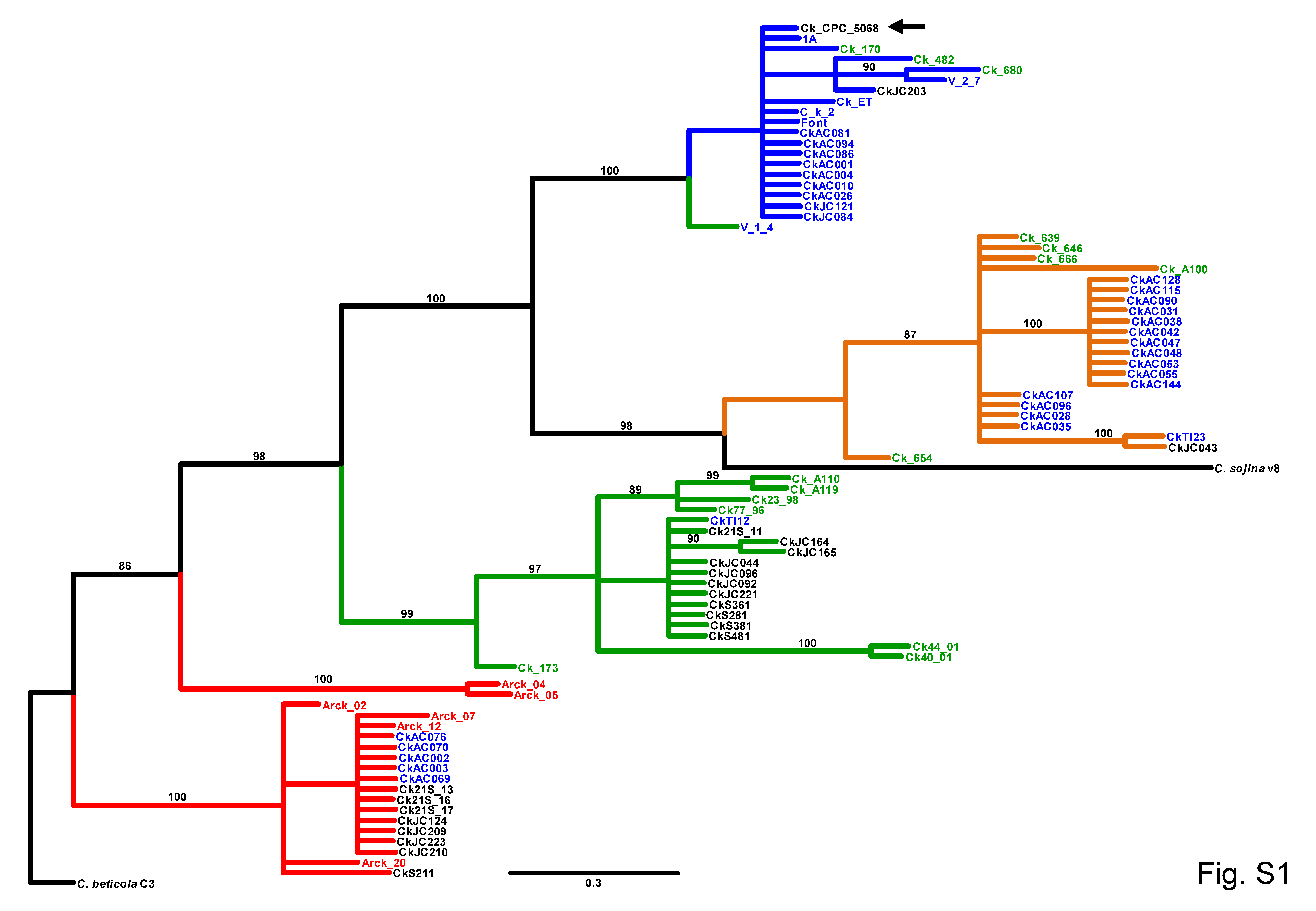

Supplement: S1 Fig — The dataset was 714 bases. Cercospora sojina is shown for reference purpose; the tree was rooted in C. beticola. Branch lengths are drawn to scale; nodal support values are given as posterior probabilities (%) above the branches (when ≥85%). Scale bar corresponds to the expected number of substitutions per site. Color in ingroup terminals according to the origin of the isolates: blue, Argentina; green, Brazil; red, United States; black, Japan. Color in ingroup branches according to lineage: blue, lineage 1; green, lineage 2; orange, lineage 3; and red, lineage 4. Black arrow indicates the ex-type strain of C. kikuchii (CPC_5068). (TIFF) [file pone.0133495.s001.tiff]
