# Supplementary material for: More Cercospora Species Infect Soybeans across the Americas than Meets the Eye
Source: PLoS One. 2015 Aug 7;10(8):e0133495. doi: 10.1371/journal.pone.0133495 (PMC4529236; doi:10.1371/journal.pone.0133495)
Supplement: S1 Table — (PDF) [file pone.0133495.s002.pdf]

S1 Table. Detailed information on PCR primers used in this study.

Target genes	Primer pairs	Ta*	Amplicon size (bp)	Reference
act (actin)	ACT-512F (5′–ATGTGCAAGGCCGGTTTCGC–3′)	53	226	[6]
	ACT-783R (5′–TACGAGTCCTTCTGGCCCAT–3′)			[6]
cfp (cercosporin facilitator protein)	F1771 (5'–ATTGGTTGGAGCGACTTTTG–3')	56	935	This work
	R2706 (5'–CAGGACAGTCTGGACAGCAA–3')			This work
	cfp_F1 (5'–GCCGATCGATTGCAGGAGTTGGC–3')	58	696	This work
	cfp_R1 (5'–TTGCTGATCCAAGTAGTCGGACG–3')			This work
cal (calmodulin)	CAL-228F (5′–GAGTTCAAGGAGGCCTTCTCCC–3')	56	561	[6]
	CAL-737R (5′–CATCTTTCTGGCCATCATGG–3')			[6]
his (histone H3)	CYLH3F (5′–AGGTCCACTGGTGGCAAG–3')	57	412	[6]
	CYLH3R (5′–AGCTGGATGTCCTTGGACTG–3')			[6]
ITS (internal transcribed spacers +5.8S nrDNA)	ITS1 (5′–TCCGTAGGTGAACCTGCGG–3′)	51	560	[6]
	ITS4 (5′–TCCTCCGCTTATTGATATGC–3′)			[6]
tef (translation elongation factor 1-alpha)	TEF_F1 (5'–CCGGCAAGTCGACAACCACCG–3')	57	605	This work
	TEF_R1 (5'–TCACGGTGACCTGGGGCGTC–3')			This work
tub (β-tubulin 1)	Ck_Betatub-F1 (5'–TGCGTGAAATCGTGAGTCCTCAC–3')	67	538	This work
	Bt516R (5′–ACGAAGTTGTCTGGGCGGAAAAG–3′)			[25]
	Tub-F1 (5′–GGTCATTACACTGAGGGTGC–3′)	56	834	[30]
	Tub-R1 (5′–GACAAGATCGTTCATGTTGGACTC–3′)			[30]
cyb (mitochondrial cytochrome b)	Cytb-F2 (5'–GGAAGAGGTCTATACTATGG–3')	45	696	[28]
	Cytb-R1 (5′–CGTGATTAGCACCTAATTGC–3′)			[28]

*Ta = Annealing temperature (^{o}C)
